# The Eysenck Personality Questionnaire Revised – Abbreviated (EPQR-A): psychometric properties of the Brazilian Portuguese version

**DOI:** 10.47626/2237-6089-2021-0342

**Published:** 2023-02-02

**Authors:** Victória Machado Scheibe, Augusto Mädke Brenner, Gianfranco Rizzotto de Souza, Reebeca Menegol, Pedro Armelim Almiro, Neusa Sica da Rocha

**Affiliations:** 1 Faculdade de Medicina Universidade Luterana do Brasil Canoas RS Brazil Faculdade de Medicina, Universidade Luterana do Brasil, Canoas, RS, Brazil.; 2 Centro de Pesquisa Clínica Hospital de Clínicas de Porto Alegre Porto Alegre RS Brazil Centro de Pesquisa Clínica, Hospital de Clínicas de Porto Alegre, Porto Alegre, RS, Brazil.; 3 Grupo de Pesquisa em Inovações e Intervenções em Qualidade de Vida UFRGS Porto Alegre RS Brazil Grupo de Pesquisa em Inovações e Intervenções em Qualidade de Vida, Universidade Federal do Rio Grande do Sul (UFRGS), Porto Alegre, RS, Brazil.; 4 Faculdade de Medicina Universidade Federal de Ciências da Saúde de Porto Alegre Porto Alegre RS Brazil Faculdade de Medicina, Universidade Federal de Ciências da Saúde de Porto Alegre, Porto Alegre, RS, Brazil.; 5 Faculdade de Psicologia UFRGS Porto Alegre RS Brazil Faculdade de Psicologia, UFRGS, Porto Alegre, RS, Brazil.; 6 Centro de Pesquisa em Psicologia Universidade Autónoma de Lisboa Lisboa Portugal Centro de Pesquisa em Psicologia, Universidade Autónoma de Lisboa, Lisboa, Portugal.; 7 Departamento de Psiquiatria UFRGS Porto Alegre RS Brazil Departamento de Psiquiatria, UFRGS, Porto Alegre, RS, Brazil.; 8 Programa de Pós-Graduação em Psiquiatria e Ciências do Comportamento UFRGS Porto Alegre RS Brazil Programa de Pós-Graduação em Psiquiatria e Ciências do Comportamento, UFRGS, Porto Alegre, RS, Brazil.

**Keywords:** Personality assessment, psychometrics, P-E-N model, confirmatory factor analysis, validation study, Brazil

## Abstract

**Introduction:**

The Eysenck Personality Questionnaire Revised – Abbreviated (EPQR-A) consists of 24 items for assessment of the three fundamental personality traits (psychoticism, extraversion, and neuroticism) and a validity scale (lie scale). Our objectives were to assess the psychometric properties of a version of this instrument culturally adapted for Brazil.

**Method:**

321 participants were recruited using a non-probabilistic method.

**Results:**

Internal consistencies ranged from minimally acceptable to respectable, except for the psychoticism domain. Higher neuroticism scores were associated with higher depression and anxiety scores, higher extraversion scores were associated with lower levels of depression symptoms, and higher psychoticism scores were associated with higher levels of depression symptoms.

**Conclusion:**

Our findings describe sustainable psychometric properties for the Brazilian Portuguese version of EPQR-A.

## Introduction

According to Eysenck, personality can be defined as a more or less stable and enduring organization of a person’s character (conative behavior system), temperament (affective behavior system), intellect (cognitive behavior system), and physique (bodily configuration and neuroendocrine endowment), which determines their unique adjustment to the environment.^1^ Eysenck’s personality model considers the existence of what he called superfactors, dimensions, or traits. According to this model, the three fundamental dimensions of personality are psychoticism (P), extraversion (E), and neuroticism (N). Each of these dimensions is expressed in terms of a continuum, and people can be classified at any point of the scales, from extremes to median points.^2^

In the E dimension, people are shy and retracted on one side (introversion) and sociable and uninhibited on the other (extraversion). The same happens with the N dimension: the neurotic or emotionally unstable personality is located at one extreme and the emotionally stable personality lies at the other. Individuals with high N scores are overly emotional, anxious, and depressed, frequently experience feelings of guilt, have low self-esteem, and tend to suffer from psychosomatic disorders. The opposite happens with stable individuals, who are typically calm, steadfast, easygoing, and able to control their emotions. The P dimension is characterized by impulsivity on one side and impulse control on the other. The main characteristics of high P scores are hostility, cruelty, lack of empathy, and non-conformism. Eysenck believed that high levels of P are linked to increased vulnerability to psychosis and considered that the biological bases of personality could provide an explanation for certain behaviors through the physiological functioning of the central nervous system.^2 , 3^

The Eysenck Personality Questionnaire (EPQ) contains 90 items^4^ and the Revised Eysenck Personality Questionnaire (EPQ-R) contains 100 items.^3^ The EPQ has the same factorial structure as the EPQ-R, but the original version had some psychometric limitations related to the P domain and the revised version of the questionnaire was the result of efforts to fix this problem. Reliability indices for the new P domain were improved and achieved acceptability, but were nevertheless not as high as those for the other domains. It should however be remembered that the P scale explores characteristics such as hostility, cruelty, little evidence of socialization, and lack of empathy, which may cause the lower reliability levels.^3^

One of the consequences of the continuous development and improvement of these scales was a progressive increase in questionnaire size. This increase can be explained by the introduction of additional personality items forming a lie/social desirability scale (L)^3^ – to facilitate detection of faking – and by the psychometric principle that larger size increases questionnaire reliability.^5 , 6^ Although longer tests measure constructs more accurately, there are certain practical disadvantages to using them. There are numerous situations in which a research project would benefit from inclusion of a personality measure, but 90 or 100 items of additional information would increase the general questionnaire to an undesirable size. In contrast, shorter tests, even those with reliable psychometric properties, inevitably have a more limited measurement capability, but can be more easily incorporated into longer assessment protocols, complementing the data obtained.

Eysenck et al.^3^ developed a short form of the EPQ-R, the Eysenck Personality Questionnaire Revised – Short Form (EPQR-S), for use in adults. In this version, the four subscales (the N, E, and P dimensions and the L scale) each contain 12 items, making a total of 48 items. The authors reported reliability for men and women, respectively, of 0.84 and 0.80 for N, 0.88 and 0.84 for E, 0.62 and 0.61 for P, and 0.77 and 0.73 for social desirability (L). Although the EPQR-S was developed explicitly “for use when time is very limited,” some might still consider the 48-item questionnaire too long and cease using personality variables in their research for reasons of convenience.^3^

The Eysenck Personality Questionnaire Revised – Abbreviated (EPQR-A) is a 24-item inventory comprising four 6-item subscales (E, N, P, and L) that was developed for researchers to use when time is limited.^7^ Researchers administered all of the EPQ and EPQR-S items to a sample of 685 undergraduate students in England, Canada, and Australia,^3 , 4^ analyzing the data using item-total correlations for each of the four subscales of the EPQR-S. The six items with the highest item-total correlations for each of the subscales were selected for inclusion in the EPQR-A. The reliability of EPQR-A subscales was demonstrated by internal consistency levels. Satisfactory levels of internal reliability were found for the E (0.74-0.84), N (0.70-0.77), and L (0.59-0.65) subscales. However, unsatisfactory levels were found for the P scale (0.33-0.52). Concurrent validation of the EPQR-A subscales was performed by examining their association with the original EPQ subscales. The correlations between the two forms of measuring E, N, and L ranged from 0.84 to 0.90. A considerably lower correlation was found between the two P scales (0.44-0.52).^7^

The EPQ has been adapted to Portuguese, and validated for the Brazilian population. The validation study compared personality structures in Brazilian and English men and women. The sample consisted of 636 Brazilian men and 760 Brazilian women, who were compared to 500 English men and 500 English women. Authors found that identical factors emerged for both the English and Brazilian populations and that intercorrelations of reliability scales were similar for both groups.^8^

The Portuguese version of the EPQ-R consists of 70 items with dichotomous responses distributed over four scales: N (23 items), E (20 items), P (nine items), and L (18 items). The values obtained for internal consistency were: N (0.87, “very good”), E (0.83, “very good”), P (0.55, “unacceptable”), and L (0.78, “respectable”), according to the criterion established by DeVellis.^9^ Test-retest reliability showed adequate (“very good” to “respectable”) temporal stability over four to eight weeks (according to the same criterion): N (0.86), E (0.89), P (0.72), and L (0.86).^10^ Psychometric studies of the EPQ-R suggest adequate psychometric properties when using both Classical Test Theory and Item Response Theory.^11 , 12^

Although some of these instruments, such as the EPQ (90 items), have been studied in the Brazilian population,^7^ the main psychometric properties of the EPQR-A (24 items) have not yet been evaluated. Moreover, other instruments for personality assessment that have been validated, such as the Personality Inventory for the 5th edition of the Diagnostic and Statistical Manual of Mental Disorders (DSM-V) (PID-5) (220 items)^13^ and the Dimensional Clinical Personality Inventory 2 (IDCP-2) (206 items),^14^ are composed of many items, limiting their use in long research protocols. Even the other versions of Eysenck’s questionnaires are still lengthy, which may restrict their usage in studies and hinder data collection. Thus, our objectives were to validate this brief instrument for measurement of personality dimensions so that it can be easily incorporated into longer assessment protocols, culturally adapting a Brazilian Portuguese version of the EPQR-A and assessing its psychometric properties in terms of convergent, discriminant, and construct validity. We chose a depression scale (Patient Health Questionnaire [PHQ-9]) and an anxiety scale (Generalized Anxiety Disorder [GAD-2]) to investigate evidence of the validity of the N scale of our instrument, considering that the relationship of neuroticism as a predictor of depression and anxiety symptoms is well established in literature.^15 - 17^

## Materials and methods

### Participants

Our sample comprised 321 individuals, of whom 266 were women (82.9%) and 55 were men (17.1%). Ages ranged from 18 to 74, with a mean of 44.85 (standard deviation [SD] = 13.733) and a median of 44 years. Majorities of our sample were of white ethnicity (90.3%), were married or cohabiting (61.7%), had a paid occupation (63.2%), and had graduate education (57.6%).

### Measures

Depression symptoms were assessed with the PHQ-9 and anxiety symptoms were assessed using the GAD-2 questionnaire. Personality dimensions were assessed using the EPQR-A.

### PHQ-9

The PHQ-9 is a nine-item scale that assesses the intensity and degree of incapacitation of nine depression symptoms according to the depression criteria described in the DSM-V.^18 , 19^ Each item has four possible answers (not at all, several days, more than half the days, and nearly every day), scored from 0 to 3 points. A depression episode was defined as presence of five or more items of the PHQ-9, at least one of which was the first or second item. The Cronbach’s alpha coefficient for the PHQ-9 was 0.88 in our sample.

### GAD-2

The GAD-2 is a two-item questionnaire on the anxiety symptoms “feeling nervous, anxious, or on edge” and “not being able to stop or control worrying.”^20^ Each item has four possible answers (not at all, several days, more than half the days, and nearly every day), scored from 0 to 3 points. Generalized anxiety was defined as when the participant’s GAD-2 score (sum of the scores for both items) was greater than or equal to 3. The Cronbach’s alpha coefficient for the GAD-2 was 0.86 in our sample.

### EPQR-A

The EPQR-A consists of 24 items divided into four scales: N, E, P, and L. Each scale has six items, each of them with a dichotomous response format (yes or no); each answer is scored specifically, according to the scale, as 0 or 1. Scoring for each question is predetermined because some questions have reverse coding.^7^

### Procedures

Participants were recruited by sharing the research protocol via social media in a non-probabilistic method. We conducted an online self-report survey in order to avoid spread of the severe acute respiratory syndrome coronavirus 2 (SARS-CoV-2). Data on age, gender, education, marital status, ethnicity, occupation, depression and anxiety symptoms, and personality were collected from July to August of 2020.

The online questionnaire was presented in Google Forms (Google, Mountain View, CA, USA) to facilitate participants’ access. The main social media platforms used for sharing the online form were Facebook and WhatsApp (Facebook Inc., Menlo Park, CA, USA). Participants declared they were over 18 years of age and completed an online informed consent form. All responses were anonymous and optional; each participant could stop answering or refuse to answer at any point of the questionnaire.

Our first step was to contact the instrument developer, Leslie J. Francis, in order to obtain permission to translate the EPQR-A instrument. A three-person bilingual team handled the translation process. First, one person independently produced an English to Brazilian Portuguese translation. Second, a second member of the group produced an independent back-translation from Brazilian Portuguese to English and the third member of the team produced a second independent English to Brazilian Portuguese translation. Finally, the three-person team compared the two Brazilian Portuguese translations and came up with a final Brazilian Portuguese version of the EPQR-A, shown in Supplementary Material S1, available online-only. Once the questionnaire was ready, a 10-person group conducted a debriefing process with the final Brazilian version. This group included a team of expert psychiatrists in quality of life questionnaires and their applications, a psychologist, and medical and psychology students. Issues of lexical and cultural equivalence of the questionnaire items were discussed. The questionnaire’s stages are shown in Supplementary Material S2 (online-only).

### Statistical analyses

Descriptive data were presented using sums, means, SDs, ranges, and percentages. Our analyses were based on assessment of the questionnaire’s construct validity (measured by confirmatory factor analysis [CFA]), convergent validity (evaluated using Pearson’s correlation coefficients), discriminant validity (assessed with *t* tests for independent samples), and reliability (assessed in terms of internal consistency). Internal consistency was evaluated by calculating Cronbach’s alpha and McDonald’s omega for each of the EPQR-A domains.^21^ Normality was assessed with the Kolmogorov-Smirnov test. However, due to our sample size, all variables were considered parametric.^22^ The *t* test for independent samples was used to compare EPQR-A domains between gender and age groups. The age variable was dichotomized using its median value for the independent samples *t* test. We calculated Cohen’s d for sample effect size when performing the *t* test and interpreted the results according to the Marôco criteria.^23 , 24^ We used Pearson’s bivariate correlation test to evaluate the correlation coefficients between EPQR-A domains and PHQ-9 and GAD-2 scores. The *t* test for independent samples was also used to compare mean EPQR-A domain scores between depressed and non-depressed subjects and anxious and non-anxious individuals. Furthermore, linear regression was used to assess whether there were linear relationships between EPQR-A domains and gender (coded 0 for female and 1 for male), age, PHQ-9 scores, and GAD-2 scores.

Confirmatory factor analysis was used to examine the factor structure of the Brazilian EPQR-A (construct validity). The four-factor model (N, E, P, L) assessed by the EPQR-A was estimated using the maximum likelihood method, which is a well-established factor structure for the PEN model of personality measures.^12 , 25 , 26^ As Furnham et al.^25^ concluded: “the EPQ factors are strongly replicable across all 34 countries; that is, the original UK data can be replicated using data from any countries.” The goodness-of-fit indices considered were: chi-square (χ^2^); ratio of chi-square to degrees of freedom (χ^2^/df); comparative fit index (CFI); Tucker-Lewis index (TLI); standardized root mean square residual (SRMR); and root mean square error of approximation (RMSEA). A confirmatory model has a good fit when the ratio χ^2^/df < 3, CFI > 0.95 (CFI > 0.90 is acceptable), TLI > 0.95 (TLI > 0.90 is acceptable), SRMR < 0.08, and RMSEA < 0.06.^27 - 29^ Statistical significance was defined at a 95% confidence interval (95%CI). All statistical analyses were performed using IBM SPSS Statistics version 22.0 (Armonk, NY, 2020), IBM SPSS Amos version 20.0 (Arbuckle, 2011) and JASP version 0.14.1 (University of Amsterdam, 2020).

### Ethical statement

The present study was approved by the ethics committee at the Hospital de Clínicas de Porto Alegre, in Porto Alegre, southern Brazil, and by the Brazilian National Committee of Research Ethics under CAAE no. 30487620.7.0000.5327, in accordance with the Declaration of Helsinki.

## Results

### Factor analysis

**Figure 1:**
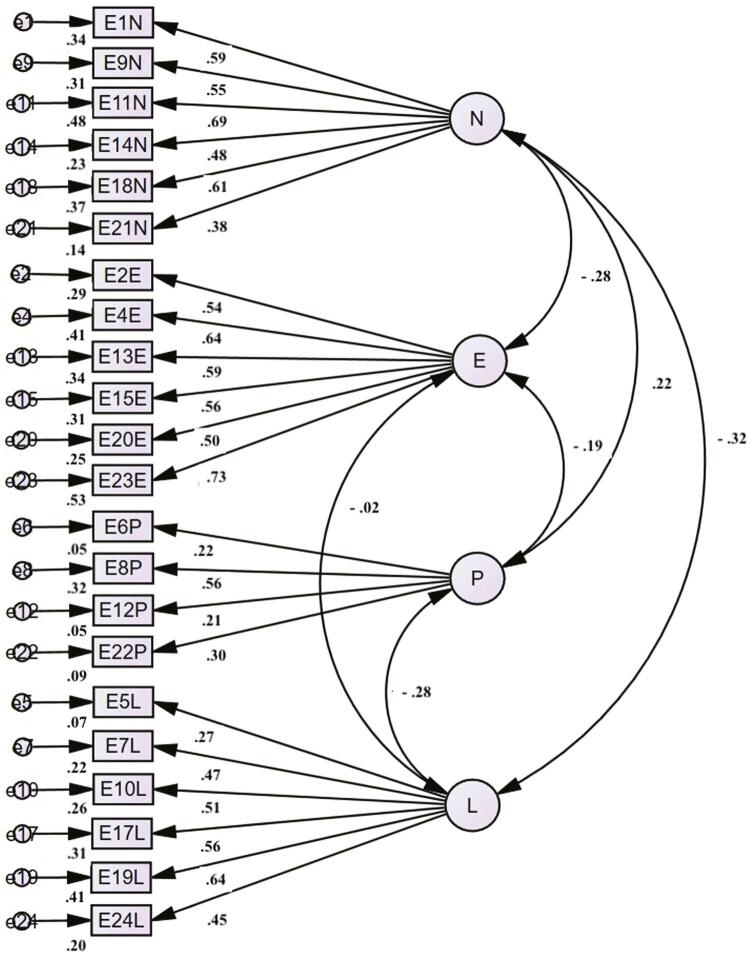
Internal consistency (reliability).

The baseline model of the four latent variables of the EPQR-A yielded the following goodness-of-fit indices in our sample: χ^2^_(246)_ = 354.025, p < 0.001; χ^2^/df = 1.44; CFI = 0.895; TLI = 0.882; SRMR = 0.059; RMSEA = 0.037. However, items 3 and 16 of the P scale had factor loadings which were less than acceptable. Therefore, the confirmatory model was retested excluding these two items and the goodness-of-fit indices showed a small improvement, yielding χ^2^_(203)_ = 305.143, p < 0.001; χ^2^/df = 1.27; CFI = 0.900; TLI = 0.886; SRMR = 0.059; and RMSEA = 0.040. Figure 1 shows the path diagram for the EPQR-A CFA model tested (standardized solution). Internal consistencies for each EPQR-A domain are shown in Table 1 . Cronbach’s alpha coefficients were 0.719 for the N domain, 0.766 for E, 0.225 for P, and 0.651 for L.

**Table 1:** Reliability coefficients (Cronbach’s alpha and McDonald’s omega) for each domain of the EPQR-A questionnaire

Domain item	n (%)	Mean (SD)	Omega	Alpha
Neuroticism	317 (98.8)	2.82 (1.806)	0.721	0.719
1	317	0.44 (0.497)		
9	317	0.56 (0.498)		
11	317	0.42 (0.494)		
14	317	0.80 (0.397)		
18	317	0.22 (0.413)		
21	317	0.39 (0.489)		
Extraversion	318 (99.1)	3.27 (1.960)	0.766	0.766
2	318	0.67 (0.470)		
4	318	0.66 (0.474)		
13	318	0.30 (0.460)		
15	318	0.46 (0.499)		
20	318	0.61 (0.489)		
23	318	0.57 (0.496)		
Psychoticism	319 (99.4)	0.79 (0.868)	0.332	0.225
3	319	0.04 (0.191)		
6	319	0.11 (0.309)		
8	319	0.19 (0.396)		
12	319	0.10 (0.297)		
16	319	0.01 (0.097)		
22	319	0.34 (0.475)		
Lie scale	316 (98.4)	4.40 (1.555)	0.666	0.651
5	316	0.95 (0.226)		
7	316	0.74 (0.437)		
10	316	0.57 (0.495)		
17	316	0.71 (0.455)		
19	316	0.72 (0.447)		
24	316	0.70 (0.459)		

EPQR-A = Eysenck Personality Questionnaire Revised – Abbreviated; SD = standard deviation.

### Discriminant validity

There were no significant differences between mean subscale values when compared between genders, except for N ( *t* = -3.363; p < 0.01; d = 0.52 [large effect]), as shown in Table 2 . Moreover, no significant differences were observed between mean values in the comparison between age groups, except for N ( *t* = -4.271; p < 0.001; d = 0.48 [medium effect]) and L ( *t* = 5.854; p < 0.001; d = 0.34 [medium effect]).

**Table 2:** Comparison of EPQR-A results between genders, age groups, and depressed/not depressed and anxious/not anxious subjects

Variables	Neuroticism mean (SD)	Extraversion mean (SD)	Psychoticism mean (SD)	Lie Scale mean (SD)
Gender*				
Male	2.09 (1.59)	3.36 (2.00)	0.98 (1.01)	4.09 (1.68)
Female	2.98 (1.81)	3.26 (1.95)	0.75 (0.83)	4.45 (1.52)
*t*	-3.363	0.348	1.838	-1.576
p	**0.001** ^‡^	0.728	0.067	0.116
d^†^	0.52	0.05	0.25	0.22
Age*				
Range	18-74	18-74	18-74	18-74
≥ 44 (n = 164)	2.41 (1.67)	3.46 (1.41)	0.72 (0.85)	4.43 (1.53)
< 44 (n = 153)	3.26 (1.84)	3.09 (1.99)	0.85 (0.88)	3.88 (1.68)
*t*	-4.271	1.685	-1.296	5.854
p	**0.000** ^§^	0.093	0.196	**0.000** ^§^
d^†^	0.48	0.21	0.15	0.34
PHQ-9*				
Depressed (n = 55)	4.64 (1.19)	2.71 (2.01)	0.93 (0.86)	4.21 (1.65)
Not depressed (n = 262)	2.44 (1.68)	3.40 (1.93)	0.76 (0.87)	4.43 (1.53)
*t*	-9.215	2.326	-1.332	0.918
p	**0.000** ^§^	**0.017** ^||^	0.188	0.337
d^†^	1.51	0.35	0.20	0.14
GAD-2*				
Anxious (n = 93)	3.93 (1.46)	2.88 (1.99)	0.96 (0.95)	4.27 (1.59)
Not anxious (n = 221)	2.35 (1.73)	3.47 (1.92)	0.72 (0.82)	4.46 (1.54)
*t*	-7.748	2.421	-2.147	1.003
p	**0.000** ^§^	**0.014** ^||^	**0.024** ^||^	0.310
d^†^	0.98	0.30	0.27	0.12

EPQR-A = Eysenck Personality Questionnaire Revised – Abbreviated; GAD = Generalized Anxiety Disorder; PHQ = Patient Health Questionnaire.

* Evaluated with an independent samples *t* test; ^†^ Cohen’s d; ^‡^ p < 0.01; p < 0.001; ^||^ p < 0.05.

Table 2 also presents the discriminant validity analyses using the depression and anxiety indices measured with the PHQ-9 and GAD-2 respectively as external validation criteria. In a comparison between depressed and not depressed individuals, as assessed by the PHQ-9, there were significant differences between mean values for N ( *t* = -9.215; p < 0.001; d = 1.51 [very large effect]) and for E ( *t* = 2.392; p < 0.05; d = 0.35 [medium effect]). Furthermore, when comparing anxious and not anxious individuals (assessed by the GAD-2 scale), mean values were significantly different for N ( *t* = -7.748; p < 0.001; d = 0.98 [large effect]), E ( *t* = 2.459; p < 0.05; d = 0.30 [medium effect]), and for P ( *t* = -2.276; p < 0.05; d = 0.27 [medium effect]).

In consonance with this study’s objectives and considering that the N dimension assesses neurotic personality traits such as depression, anxiety, and emotional instability, our results indicated the proximity of constructs evaluated by the PHQ-9 and GAD-2 instruments, thus demonstrating the validity of the EPQR-A N scale. It is important to emphasize that this is the most useful scale in the context of psychopathology and mental health assessment, since the other scales (E, P, L) have weaker correlations, although they are in the expected direction.

### Convergent validity

The correlations (Pearson’s correlation coefficient [ *r* ]) between EPQR-A domains and PHQ-9 scores were all significant (N, E, and L: p < 0.001; P: p < 0.01) and the coefficient between N and PHQ-9 had a large effect according to Cohen’s criterion.^23^ Positive correlations were found for the N ( *r* = 0.639; p = 0.000) and P domains ( *r* = 0.164; p = 0.003), which indicated that these domains and PHQ-9 scores tended to vary in the same direction. On the other hand, negative correlations were found for E ( *r* = -0.225; p = 0.000) and L ( *r* = -0.247; p = 0.000). Moreover, the correlations between the EPQR-A N, E, and L domains and GAD-2 scores were found to be significant (p < 0.001, < 0.01, and < 0.05, respectively), and that between N and GAD-2 had a large effect according to the same criterion. N had a positive correlation with the GAD-2 score ( *r* = 0.562; p = 0.000), whereas E ( *r* = -0.157; p = 0.005) and L ( *r* = -0.138; p = 0.015) had negative correlations.

### Multiple linear regression

The linear relationships between EPQR-A domains and gender (coded 0 for female and 1 for male), age, PHQ-9 score, and GAD-2 scores assessed with multiple linear regression are shown in Table 3 . The N domain appeared to have a negative linear relationship (β = -0.186) with gender, meaning higher N scores were associated with female participants. In addition, the N domain had a negative relationship with age (β = -0.161), since higher N scores were found in younger participants. Furthermore, the N domain showed a positive relationship with PHQ-9 and GAD-2 scores (β = 0.419 and 0.236, respectively). Higher N scores were thus associated with higher depression and anxiety scores, as expected. The E domain showed a negative relationship with PHQ-9 scores (β= -0.221), since higher E scores were associated with lower levels of depression symptoms. The P domain was found to have a negative linear relationship with age (β = -0.122), since higher P scores were found in younger participants. Moreover, the P domain showed a positive relationship with PHQ-9 scores (β = 0.194), i.e., higher P scores were associated with higher levels of depression symptoms. The L domain was found to have a positive linear relationship with age (β = 0.351): higher L scores were found in older participants, as demonstrated by previous studies.^30 , 31^ Finally, the L domain showed a negative relationship with PHQ-9 (β = -0.215), indicating that higher L scores were associated with lower levels of depression symptoms.

**Table 3:** Linear regression analyses for relationships between EPQR-A domains and sex, age, anxiety symptoms, and depression symptoms

EPQR-A domains	Beta	Standardized Beta	R^2^	p-value
Neuroticism				
Gender*	-0.886	-0.186		**0.001** ^†^
Age	-0.021	-0.161	0.467	**0.000** ^‡^
PHQ-9	0.127	0.419		**0.000** ^‡^
GAD-2	0.251	0.236		**0.000** ^‡^
Extraversion				
Gender*	0.101	0.020		0.728
Age	0.000	0.001	0.051	0.981
PHQ-9	-0.073	-0.221		**0.005** ^†^
GAD-2	-0.011	-0.010		0.899
Psychoticism				
Gender*	0.236	0.103		0.067
Age	-0.008	-0.122	0.060	**0.036** ^§^
PHQ-9	0.029	0.194		**0.013** ^§^
GAD-2	-0.028	-0.053		0.483
Lie scale				
Gender*	-0.365	-0.089		0.116
Age	0.040	0.351	0.191	**0.000** ^‡^
PHQ-9	-0.057	-0.215		**0.003** ^†^
GAD-2	0.066	0.071		0.312

EPQR-A = Eysenck Personality Questionnaire Revised – Abbreviated; GAD = Generalized Anxiety Disorder; PHQ = Patient Health Questionnaire.

* 0 = female, 1 = male; ^†^ p < 0.01; ^‡^ p < 0.001; ^§^ p < 0.05.

## Discussion

This is the first study to culturally adapt the EPQR-A for Brazil and to assess its psychometric properties in terms of reliability and validity in a Brazilian Portuguese version of the questionnaire. Our findings describe sustainable psychometric properties of the culturally adapted Brazilian Portuguese version of the EPQR-A, demonstrating the reliability and validity of the dimensions assessed by the questionnaire, even though it is imperative to highlight the low reliability of the P domain, as described in previous studies.^7^

The construct validity of the Brazilian version of EPQR-A was examined through the CFA. The four-factor model (N, E, P, L) of this personality instrument was tested and yielded adequate goodness-of-fit indices (χ^2^_[239]_ = 304.198, p < 0.001; χ^2^ /df = 1.27; CFI = 0.937; TLI = 0.927; SRMR = 0.054; RMSEA = 0.029). These results demonstrate the adequacy of the Brazilian version of the EPQR-A for assessing the neuroticism, extraversion, and psychoticism constructs and the lie/social desirability construct measured by the L scale, in the Brazilian context.

The internal consistency coefficients were similar to those reported in other studies such as the EPQR-A development study.^8 , 12^ According to DeVellis’^9^ criterion, Cronbach’s alpha values for the domains ranged from “minimally acceptable” to “respectable,” except for the P domain, which was categorized as “unacceptable.” These findings can be explained by the content of the psychological constructs (mainly the P domain) and the self-reported nature of the instrument. Field suggests that, when considering psychological constructs, a Cronbach’s alpha below the ideal cut-off of 0.7 to 0.8 may be expected as a consequence of the diversity of the constructs.^32^ Shorter scales also tend to have lower internal consistency when compared to the longer scales from where they were adapted because alpha values depend on the number of items on the scale. Therefore, Field also suggests exercising caution when interpreting alpha values because higher values can sometimes be achieved by a larger number of items and not by increasing reliability.^32^ Another explanation for the absence of highly reliable alphas is the presence of reverse phrasing items on EPQR-A. Reverse phrasing is an important part of the questionnaire because it “tests” the individual’s attention, but tends to lower Cronbach’s alpha values because it affects the average covariance between items.

The analysis of discriminant validity revealed that the EPQR-A N domain discriminated between gender and age groups, whereas the L domain could only discriminate between age groups; other questionnaire domains (E and P) were not significantly discriminative between gender and age groups. Considering depressed and anxious individuals, N was the only EPQR-A domain that discriminated between depressed and not depressed and between anxious and not anxious individuals. This finding suggests that N scores are significantly different between depressed and not depressed people (or anxious and not anxious). The other domains were not significantly discriminative. Similar results were reported in studies with unipolar and bipolar depressed patients. They found that unipolar patients had higher N scores when compared to healthy controls.^33 , 34^

Findings of convergent validity between the EPQR-A domains and the PHQ-9 questionnaire were all significant. The N and P domains had positive correlations with PHQ-9 scores, whereas E and L had negative correlations. These findings suggest that individuals with higher N and P scores also tended to have higher depression scores. Moreover, individuals with higher E and L scores thus tended to have lower depression scores. In a study on neuroticism and victimization in childhood, increased PHQ-9 and N scores were observed among patients with major depressive disorder when compared to healthy controls.^35^ Another study found neuroticism was a vulnerability factor for depression.^36^ Other studies showed extraversion was also significantly lower in depressed individuals.^33 , 34^ Regarding the GAD-2 questionnaire, the N, E, and L domains were found to have significant correlations with anxiety scores. The only domain with a positive correlation was N, meaning that individuals with higher N scores tended to also have higher anxiety scores, while those with higher E and L scores tended to have lower anxiety scores.

In agreement with the literature, higher N scores were associated with female participants.^37 , 38^ Therefore, male gender may be considered a protective factor for N. This information raises a series of questions on the origins of such differences, but a previous study has discussed the possible influence of socially learned gender roles over a biological basis for this distinction, particularly for the neuroticism and psychoticism traits.^37^ Moreover, younger age was related to higher N and P scores, while older age was related to higher L scores. These findings are coherent with those published by Soto et al.^39^ who found increased mean neuroticism in early life, mainly in girls, as opposed to a lower mean neuroticism in adulthood. The same findings apply to the depression and anxiety facets of neuroticism.

Use of the EPQR-A as a test that assesses the dimensions of personality enables examination of a person’s potentialities and limitations, which makes it possible to manage these characteristics towards an individual’s goals. In addition, this tool can be useful for improving human relationships, since knowing the dynamics of attitudes and functions enables people to minimize conflicts and understand different points of view. The EPQR-A is an important questionnaire for psychometric research. The lower number of questions makes it easier to apply and allows it to be combined with other scales, whereas longer versions may demand more time and patience from individuals.

As for the purpose and relevance of this study, there is a need for development of reliable and valid instruments that integrate broader protocols for psychological assessment and psychopathological symptoms. As a future perspective, studies targeting reformulation of the psychoticism items to improve the psychometric properties of this dimension could be important. However, this could impair comparability with the original scale. Furthermore, studies based on other analytical strategies focusing more on the item rather than the dimension as a whole could also be useful.

This study with the Brazilian version of EPQR-A evaluated a sample of Brazilian adults that is not necessarily representative of the general population. In addition, answers to items on the P scale often suffer from bias caused by the behavior of L, which is more evident when the scale is smaller, as in the present case.

More studies are needed in different settings due to the cultural, economic, and social diversity of the Brazilian population. Recognizing the need for prospective longitudinal studies in this field is crucial in order to further qualify the assessment of patients’ behavioral and emotional characteristics, aiming to quantify and describe personality dimensions and traits in different samples.

## Conclusion

The EPQR-A questionnaire could become a reference tool for studies on personality assessment, providing an extensive description of patients’ behavioral and emotional characteristics. The EPQR-A questionnaire adapted for Brazilian culture has produced results that reveal satisfactory equivalence to the original version and suggest that this is a reliable and valid option for evaluating personality dimensions in the Brazilian population, although it is important to emphasize the low reliability of the P domain.
