# Corrigendum

**DOI:** 10.47626/2237-6089-2023-0001

**Published:** 2023-05-02

We hereby declare that three errors were detected after publication of the article titled “The Eysenck Personality Questionnaire Revised – Abbreviated (EPQR-A): psychometric properties of the Brazilian Portuguese version” (doi: https://doi.org/10.47626/2237-6089-2021-0342), in Trends in Psychiatry and Psychotherapy volume 45 (e20210342).

First, affiliation no. 6 should read “Centro de Investigação em Psicologia” (rather than “Centro de Pesquisa em Psicologia”).

Second, the legend of Figure 1 should read “Path diagram for the EPQR-A CFA model” (rather than “Internal consistency”).

Finally, the second subtitle of the Results section, “Internal consistency”, had been erroneously omitted and presented as figure legend.

