# Supplementary material for: The Eysenck Personality Questionnaire Revised – Abbreviated (EPQR-A): psychometric properties of the Brazilian Portuguese version
Source: Trends Psychiatry Psychother. 2023 Feb 2;45:e20210342. doi: 10.47626/2237-6089-2021-0342 (PMC9991414; doi:10.47626/2237-6089-2021-0342)
Supplement: Supplementary file 1 [file 2238-0019-trends-45-e20210342-suppl01.pdf]

http://doi.org/10.47626/2237-6089-2021-0342

Supplementary Material S1

The Portuguese Version of the Eysenck Personality Questionnaire Revised - Abbreviated (EPQR-A)

EPQR-A	SIM	NÃO
1. O seu humor varia para cima e para baixo frequentemente?	1	0
2. Você é uma pessoa falante?	1	0
3. Estar em débito (dívida) preocuparia você?	0	1
4. Você é bastante animado (entusiasmado)?	1	0
5. Alguma vez você se vangloriou por pegar algo a mais do que sua parte?	0	1
6. Você usaria drogas que podem ter efeitos estranhos ou perigosos?	1	0
7. Alguma vez você já culpou alguém por algo que você sabia que era sua culpa?	0	1
8. Você prefere fazer as coisas do seu jeito ao invés de agir de acordo com as regras?	1	0
9. Você se sente de "saco cheio" frequentemente?	1	0
10. Você já pegou algo (mesmo um alfinete ou botão) que pertencia a outra pessoa?	0	1
11. Você se considera uma pessoa nervosa?	1	0
12. Você acha que os casamentos estão fora de moda e que deveriam acabar?	1	0
13. Você consegue animar uma festa chata facilmente?	1	0
14. Você é uma pessoa preocupada?	1	0
15. Você tende a ficar em segundo plano em situações sociais?	0	1
16. Você se preocupa se sabe que há erros em seu trabalho?	0	1
17. Alguma vez você já trapaceou em um jogo?	0	1
18. Você sofre dos "nervos"'?	1	0
19. Alguma vez você já levou vantagem sobre alguém?	0	1
20. Você é mais quieto quando está com os outros?	0	1
21. Você se sente solitário frequentemente?	1	0
22. É melhor seguir as regras da sociedade do que fazer do seu próprio jeito?	0	1
23. As pessoas pensam em você como sendo muito animado (entusiasmado)?	1	0
24. Você sempre pratica aquilo que você prega?	1	0

Citation: Scheibe, V. M., Brenner, A. M., Souza, G. R., Menegol, R., Rocha, N. S. The Eysenck Personality Questionnaire Revised - Abbreviated (EPQR-A): Psychometric Properties of the Brazilian Portuguese Version. Trends in Psychiatry and Psychotherapy, 2021.

Interpretation: The EPQR-A scores are composed by each scale: Neuroticism, Extroversion, Psychoticism and Lie Scale.

Neuroticism items = item 1 + item 9 + item 11 + item 14 + item 18 + item 21
Scores range (0-6)
Extroversion items = item 2 + item 4 + item 13 + item 15 + item 20 + item 23
Scores range (0-6)
Psychoticism items = item 3 + item 6 + item 8 + item 12 + item 16 + item 22
Scores range (0-6)
Lie Scale items = item 5 + item 7 + item 10 + item 17 + item 19 + item 24
Scores range (0-6)

Each domain score is composed by the sum of each item value and ranges from 0 to 6.

http://doi.org/10.47626/2237-6089-2021-0342
The Eysenck Personality Questionnaire Revised – Abbreviated (EPQR-A): psychometric properties of the Brazilian Portuguese version - Scheibe et al.

Supplementary Material S2

Questionnaire stages

Original	First Translation	Back-Translation	Second Translation	Final Version	Post-Debriefing Version
Does your mood often go up and down?	O seu humor vai para cima e para baixo?	Does your mood goes up and down?	O seu humor varia para cima e para baixo?	O seu humor varia para cima e para baixo frequentemente?	O seu humor varia para cima e para baixo frequentemente?
Are you a talkative person?	Você é uma pessoa falante?	Are you a talkative person?	Você é uma pessoa falante?	Você é uma pessoa falante?	Você é uma pessoa falante?
Would being in debt worry you?	Estar em débito preocupa você?	Does being in debt worry you?	Ficar em dívida preocupa você?	Estar em débito preocuparia você?	Estar em débito (dívida) preocuparia você?
Are you rather lively?	Você é bastante animado?	Are you really excited?	Você é realmente animado?	Você é bastante animado (entusiasmado)?	Você é bastante animado (entusiasmado)?
Were you ever greedy by helping yourself to more than your share of anything?	Alguma vez você se envaideceu por se servir mais do que devia de algo?	Have you ever flattered yourself by serving more than you should on something?	Você já lisonjeou a si mesmo por servir mais do que devia de algo?	Alguma vez você se envaideceu por pegar a mais de algo?	Alguma vez você se vangloriou por pegar algo a mais do que sua parte?
Would you take drugs which may have strange or dangerous effects?	Você usaria drogas que podem ter efeitos estranhos ou perigosos?	Would you use drugs that can have strange or dangerous effects?	Você usaria drogas que podem ter feitos estranhos ou perigosos?	Você usaria drogas eu podem ter efeitos estranhos ou perigosos?	Você usaria drogas eu podem ter efeitos estranhos ou perigosos?
Have you ever blamed someone for doing something you knew was really your fault?	Alguma vez você culpou alguém por algo que você sabia que era seu erro?	Have you ever blamed someone for something you knew that was your mistake?	Você já culpou alguém por algo que você sabia que foi sua culpa?	Alguma vez você já culpou alguém por algo que você sabia que era a sua culpa?	Alguma vez você já culpou alguém por algo que você sabia que era sua culpa?
Do you prefer to go your own way rather than act by the rules?	Você prefere seguir da sua maneira ao invés de agir de acordo com as regras?	Do you prefer to go your own way rather than act according to rules?	Você prefere fazer do seu jeito ao invés de agir de acordo com as regras?	Você prefere fazer as coisas do seu jeito ao invés de agir de acordo com as regras?	Você prefere fazer as coisas do seu jeito ao invés de agir de acordo com as regras?
Do you often feel ‘fed-up’?	Muitas vezes você se sente de “saco cheio”?	Do you often feel fed up?	Você se sente “de saco cheio” frequentemente?	Você se sente de “saco cheio” frequentemente?	Você se sente de “saco cheio” frequentemente?
Have you ever taken anything (even a pin or button) that belonged to someone else?	Alguma vez você pegou algo (mesmo um alfinete ou botão) que pertencia a alguém?	Have you ever taken something (even a pin or a button) that belonged to someone else?	Você já tomou algo (mesmo um alfinete ou botão) que pertencia a outra pessoa?	Você já pegou algo (mesmo um alfinete ou botão) que pertencia a outra pessoa?	Você já pegou algo (mesmo um alfinete ou botão) que pertencia a outra pessoa?
Would you call yourself a nervous person?	Você se considera uma pessoa nervosa?	Do you consider yourself a nervous person?	Você se considera uma pessoa nervosa?	Você se considera uma pessoa nervosa?	Você se considera uma pessoa nervosa?
Do you think marriage is old-fashioned and should be done away with?	Você pensa que casamento é fora de moda e que deveria acabar?	Do you think that marriage is out of fashion and that it should end?	Você acha que casamentos estão fora de moda e deveriam acabar?	Você acha que casamento está fora de moda e que deveria acabar?	Você acha que casamentos estão fora de moda e que deveriam acabar?
Can you easily get some life into a rather dull party?	Você pode facilmente dar vida a uma festa meio chata?	Can you easily bring a boring party to life?	Você consegue trazer uma festa chata de volta à vida?	Você consegue trazer uma festa chata de volta à vida facilmente?	Você consegue animar uma festa chata facilmente?
Are you a worrier?	Você é uma pessoa preocupada?	Are you a concerned person?	Você é uma pessoa preocupada?	Você é uma pessoa preocupada?	Você é uma pessoa preocupada?
Do you tend to keep in the background on social occasions?	Você tende a ficar em segundo plano em situações sociais?	Do you tend to be in the background in social situations?	Você tende a estar no plano de fundo em situações sociais?	Você tende a ficar em segundo plano em situações sociais?	Você tende a ficar em segundo plano em situações sociais?

http://doi.org/10.47626/2237-6089-2021-0342

Does it worry you if you know there are mistakes in your work?	Te preocupa se você sabe que existem erros no seu trabalho?	Do you worry if you know that there are mistakes in your personal work?	Você se preocupa se você sabe que há erros em um trabalho pessoal?	Você se preocupa se você sabe que há erros em seu trabalho?	Você se preocupa se sabe que há erros em seu trabalho?
Have you ever cheated at a game?	Alguma vez você enganou alguém num jogo?	Have you ever cheated someone in a game?	Você já trapaceou de alguém em um jogo?	Alguma vez você já trapaceou em um jogo?	Alguma vez você já trapaceou em um jogo?
Do you suffer from 'nerves'?	Você sofre dos "nervos"'?	Do you suffer from "nerves"?	Você sofre dos "nervos"?	Você sofre dos "nervos"?	Você sofre dos "nervos"?
Have you ever taken advantage of someone?	Alguma vez você levou vantagem sobre alguém?	Have you ever taken advantage of someone?	Você já levou vantagem de alguém?	Alguma vez você já levou vantagem sobre alguém?	Alguma vez você já levou vantagem sobre alguém?
Are you mostly quiet when you are with other people?	Você fica mais tempo quieto quando está com os outros?	Are you more quiet when you are with others?	Você é mais quieto quando está com outros?	Você é mais quieto quando está com outros?	Você é mais quieto quando está com outros?
Do you often feel lonely?	Muitas vezes você se sente solitário?	Do you often feel lonely?	Você se sente solitário frequentemente?	Você se sente solitário frequentemente?	Você se sente solitário frequentemente?
Is it better to follow society's rules than go your own way?	É melhor seguir as regras da sociedade do que ir no seu próprio caminho?	Is it better to follow the social rules than to go your own way?	É melhor seguir as regras sociais do que fazer do seu jeito?	É melhor seguir as regras da sociedade do que fazer do seu próprio jeito?	É melhor seguir as regras da sociedade do que fazer do seu próprio jeito?
Do other people think of you as being very Lively?	As pessoas pensam em você como sendo muito animado?	Do people think of you as being really excited?	As pessoas pensam em você como realmente animado?	As pessoas pensam em você como sendo muito animado (entusiasmado)?	As pessoas pensam em você como sendo muito animado (entusiasmado)?
Do you always practice what you preach?	Você sempre pratica aquilo que você prega?	Do you Always practice what you preach?	Você sempre pratica o que prega?	Você sempre pratica aquilo que você prega?	Você sempre pratica aquilo que você prega?
